# Prodrug-based bispecific antibodies for cancer therapy: advances and future directions

**DOI:** 10.3389/fimmu.2025.1523693

**Published:** 2025-01-22

**Authors:** Zhijuan Ai, Bing Wang, Yunlong Song, Panpan Cheng, Xinlin Liu, Peng Sun

**Affiliations:** ^1^ The Affiliated Hospital of Qingdao University, Qingdao University, Qingdao, China; ^2^ Qingdao Cancer Institute, Qingdao, China; ^3^ Biomedical Center of Qingdao University, Qingdao University, Qingdao, China; ^4^ Qingdao Municipal Center for Disease Control and Prevention, Qingdao Institute of Preventive Medicine, Qingdao, China; ^5^ Department of Hepatobiliary and Pancreatic Surgery, The Affiliated Hospital of Qingdao University, Qingdao, China

**Keywords:** bispecific antibodies, prodrug, cancer therapy, mechanism, therapeutic efficacy

## Abstract

Bispecific antibodies represent an innovative paradigm in cancer therapy, offering broader therapeutic potential compared to conventional monoclonal antibodies. To increase tumor selectivity while mitigating off-target effects in normal tissues, the concept of prodrug-based bispecific antibodies has emerged. This review delineates the various mechanisms underlying the action of prodrug-based bispecific antibodies, including protease-mediated activation, steric hindrance release via proteolytic processing, activation by soluble factors, conditional assembly, and chain exchange-mediated activation. We also address the critical challenges that must be overcome to optimize the development and clinical application of these sophisticated therapeutic agents.

## 1 Introduction

Targeted cancer therapies represent a critical treatment approach; however, achieving an optimal balance of efficacy and safety remains challenging, as many targeted antigens are expressed on both tumor cells and normal tissues (1). Furthermore, effective targeted therapies are lacking for the majority of cancers, including many of the most common types. Prodrugs have emerged as a key strategy to overcome these challenges. In 1958, Albert first proposed the concept of prodrugs. A prodrug is an inactive compound that undergoes chemical or metabolic conversion to become an active therapeutic agent (2). The activation of prodrugs can be achieved through endogenous, or exogenous stimuli. Endogenous stimuli, the most commonly approved prodrug activation strategies, utilize enzymes or microenvironmental factors to activate prodrugs (3, 4). Prodrugs are extensively utilized for the targeted delivery of therapeutic agents to cancer cells. To date, prodrug-based cancer therapies have achieved significant advancements in target selection, activation chemistry, and the optimization of prodrug size and physicochemical properties (5, 6). The primary focus of prodrug application in cancer treatment lies in exploiting the biological differences between normal tissues and cancer cells, such as low pH, reactive oxygen species (ROS), or over-expressed enzymes (7). Despite their ability to enhance the selectivity and efficacy of cancer therapies, prodrugs face challenges including non-specific distribution, limited tumor penetration, and potential immunogenicity (7, 8). Furthermore, the complexity of tumor pathogenesis and evolution, coupled with the involvement of multiple mediators in signaling pathways driving tumor growth or recurrence, may constrain the efficacy of prodrug agents targeting a single molecule. Therefore, exploring innovative strategies to optimize clinical outcomes is imperative.

Bispecific antibodies (bsAbs) exhibit the unique capability to bind two distinct epitopes, either on the same or on different antigens. This dual specificity enables novel mechanisms of action (MOAs) and enhances anti-tumor activities beyond those achievable by conventional monospecific antibodies, offering extensive opportunities for therapeutic applications (9, 10). Based on their MOAs, bsAbs can be broadly categorized into the following types: dual receptor inhibition, ligand-receptor inhibition, receptor activation, and targeted payload delivery (11, 12). Clinical results have demonstrated that bsAbs can achieve potent anti-tumor activity with a favorable balance between efficacy and safety (13–15). Prodrug-based bsAbs combine the advantages of both prodrugs and bsAbs, enabling targeted delivery to specific sites where the prodrug undergoes biological conversion into active bsAbs, thereby enhancing therapeutic efficacy and minimizing off-target effects. This review will explore the MOAs underlying prodrug-based bsAbs and highlight potential strategies for their future development.

## 2 Prodrug-based bsAbs with different MOAs

### 2.1 Protease-mediated unblocking

Proteases represent one of the most abundant and diverse enzyme classes. Due to their frequent upregulation in cancerous lesions, proteases are prime candidates for the development of tumor-activated prodrugs. Examples include matrix metalloproteinase (MMP) inhibitors and cysteine cathepsins. Protease-activated bsAbs are designed as masked antibodies that are selectively activated by tumor-associated proteases within the tumor microenvironment (TME) while remaining inactive in normal tissues where protease activity is tightly controlled. Typically full-length IgG, protease-activated bsAbs are engineered using diverse masking strategies, such as (i) mimotopes and idiotypic masks; (ii) variable-domain-binding proteins extending over the paratope or disrupting its binding conformation; and (iii) nonbinding bulky moieties near the paratope to sterically hinder antigen binding (16).

One notable example is CI107, a T cell–engaging bispecific antibody (TCB) targeting both epidermal growth factor receptor (EGFR) and CD3. CI107 is designed with protease-cleavable peptide masks that block both the tumor-associated antigen-binding domain and the CD3-binding domain. Within the TME, these peptide masks are cleaved, enhancing the tumor-selective activity of the TCB. Compared to its unmasked form, dually masked CI107 showed over a 500-fold reduction in antigen binding and a 15,000-fold decrease in cytotoxic activity. Additionally, the maximum tolerated dose of CI107 in cynomolgus monkeys was more than 60 times higher than that of the unmasked TCB, with significantly reduced toxicity in treated animals (17). This strategy improves tumor-specific activity while minimizing systemic toxicity. Another bsAbs targeting EGFR and CD3 is CX-904, developed by CytomX, which is an investigational Probody therapy, designed as a masked, conditionally activated T-cell engager (TCE). CX-904 is undergoing evaluation in a phase 1 study (NCT05387265) for patients with advanced metastatic solid tumors commonly expressing EGFR. Encouraging initial signs of efficacy were observed for CX-904 in advanced pancreatic cancer, including 2 of 6 patients (33%) with a confirmed partial response (PR) and all 6 patients (100%) with disease control (**Table 1**).

**Table 1:** BsAb prodrugs in clinical investigation.

Drug	Platform/Technology	Company/Institute	Target(s)	Phase/clinical trial ID	Trial design	Efficacy	Reference
CX-904	Probody, masked	CytomX	EGFR, CD3	Phase 1/NCT05387265	CX-904 in advanced solid tumors	All 6 patients with pancreatic cancer achieved disease control. One patient (6 mg target dose) achieved an 83% tumor reduction. The second patient (5 mg target dose) with a confirmed response achieved a 51% tumor reduction	
AMX-818(SAR446309)	PRO-XTEN, protease-activated	Sanofi	HER2, CD3	Phase 1/2/NCT05356741	AMX-818 alone and in combination with pembrolizumab in locally advanced or metastatic HER2-expressing cancers		(20)
AMX-500 (SAR446329)	PRO-XTEN, protease-activated	Sanofi	PSMA, CD3	Phase 1/2/NCT05997615	AMX-500 in metastatic castration resistant prostate cancer (mCRPC)		
AMX-525 (SAR446368)	PRO-XTEN, protease-activated	Sanofi	EGFR, CD3				
TAK-186 (MVC-101)	COBRA,conditional assembly	Takeda	EGFR, CD3	Phase 1/2/NCT04844073	TAK-186 in unremovable advanced or metastatic cancer		(38)
TAK-280	COBRA,conditional assembly	Takeda	B7-H3, CD3	Phase 1/2/NCT05220098	TAK- 280 in unresectable, locally advanced or metastatic cancer		(39)

EGFR, epidermal growth factor receptor; HER2, human epidermal growth factor receptor 2; PSMA, prostate-specific membrane antigen; B7-H3, B7 homolog 3 protein.

**Figure 1:**
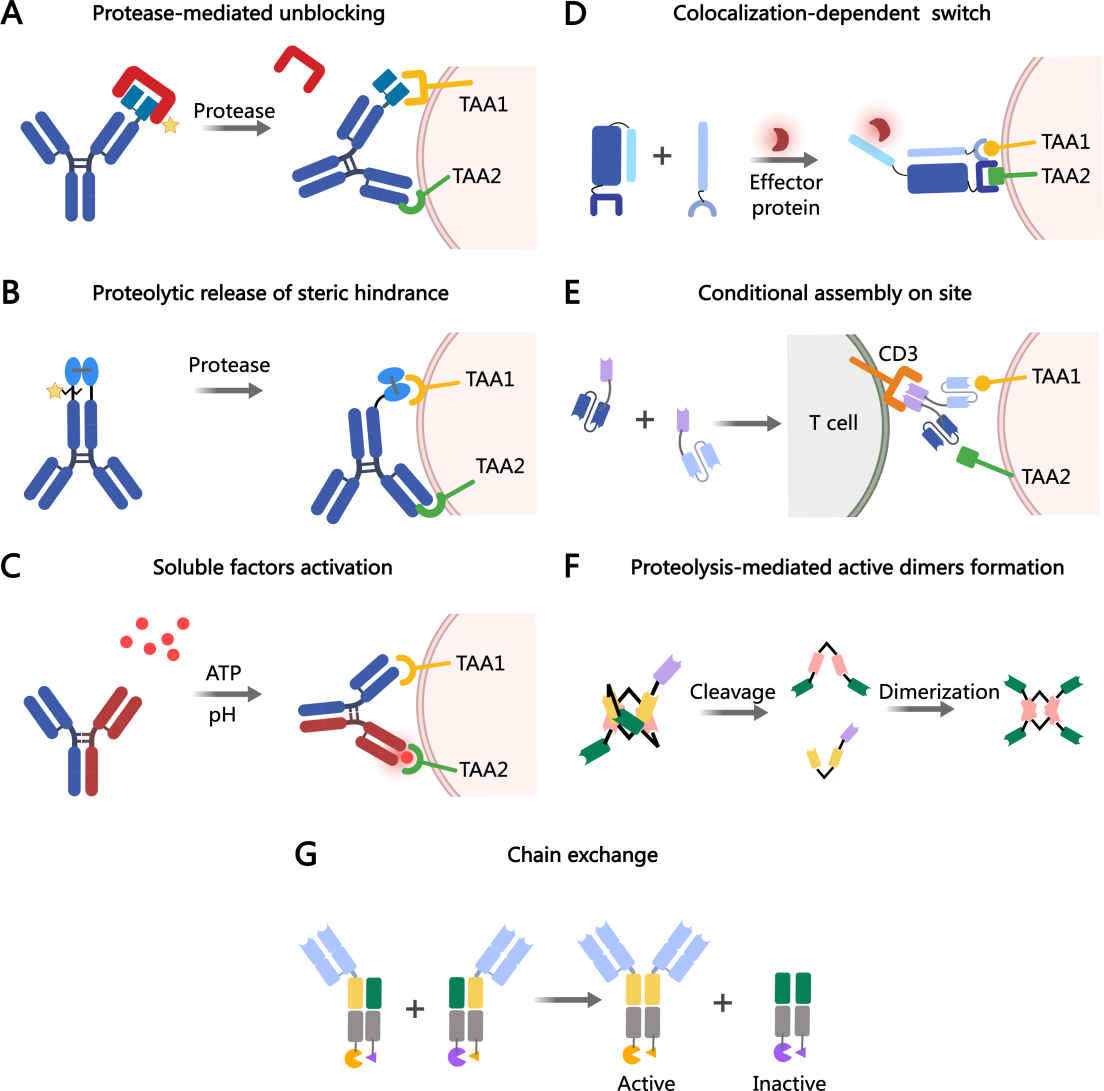
MOAs of bsAb prodrugs. **(A)** Protease-mediated activation of blocked antigen-binding sites. **(B)** Proteolytic processing restores the activation of prodrug-antibodies. **(C)** Affinity modulation is induced by the target environment (e.g., acidic pH or ATP). **(D)** Colocalization-dependent protein switch is tuned, the antibody fragments do not interact with each other, but strongly interact when colocalized on a surface by way of targeting domains. **(E)** Two antibody fragments that assemble on site. **(F)** The COBRA molecule is conditionally activated upon proteolysis in the TME, allowing for the formation of active dimers on the tumor cell. **(G)** Conditional chain-exchange mediates the reconstitution of prodrug-antibodies. The figure was created with MedPeer (medpeer.cn).

Another example is Prot-FOLR1-TCB, a protease-activated anti-folate receptor 1 TCB, equipped with an anti-idiotypic anti-CD3 mask linked to the anti-CD3 Fab via a tumor protease-cleavable linker. The potency of Prot-FOLR1-TCB is restored after protease cleavage of the linker, releasing the anti-idiotypic anti-CD3 single-chain variable fragments (scFv) (**Figure 1A**). Prot-FOLR1-TCB demonstrates *in vivo* antitumor efficacy comparable to the parental FOLR1-TCB, while the non-cleavable control remains inactive. Additionally, bronchial epithelial and renal cortical cell killing, in cells with low FOLR1 expression, is prevented compared to the parental FOLR1-TCB (18). Therefore, the specificity and safety of TCBs can be enhanced through anti-idiotypic masking of the anti-CD3 Fab fragments, with tumor-specific proteases cleaving the mask.

XPAT is a masked, precision-activated TCE protein, consisting of a TCE core with two scFvs targeting both a tumor-associated antigen (TAA) and CD3. Each scFv is connected to an XTEN mask through a protease-cleavable linker, specifically designed to be cleaved by three protease classes: matrix metalloproteinases, serine proteases, and cysteine proteases. Unmasked HER2-XPAT (uTCE) demonstrates strong cytotoxicity *in vitro*, with the XTEN polypeptide mask offering up to 4-log-fold protection. The HER2-XPAT protein induces protease-dependent antitumor activity *in vivo* and remains proteolytically stable in healthy tissues (19).

AMX-818, also known as SAR446309, designed with the PRO-XTEN masking platform, is a novel prodrug HER2-XPAT TCE exhibiting potent T cell activation and is being evaluated in a phase 1/2 study (NCT05356741) for patients with locally advanced or metastatic HER2-expressing cancers (20). Other prodrugs developed using the PRO-XTEN platform include AMX-500 (SAR446329), a dual-masked PSMA-targeted TCE, undergoing evaluation in a phase 1/2 study (NCT05997615), and AMX-525 (SAR 446368), a dual-masked EGFR-targeted TCE (**Table 1**).

The ProTriTAC platform, developed by Harpoon, comprises three humanized antibody-derived binding domains on a single polypeptide chain: anti-albumin for half-life extension, anti-CD3 for T-cell engagement, and anti-target antigen for tumor cell engagement. The anti-albumin domain, bearing a masking moiety and a protease-cleavable linker, keeps the prodrug inert by inhibiting the binding of the adjacent anti-CD3 domain to T cells. Harpoon is developing protease-activated TCE prodrugs targeting trophoblast cell surface antigen 2 (Trop2) and Integrin-β6 for the treatment of solid tumors (**Table 1**) (21, 22). Zymeworks recently introduced a PROTECT (PROgrammed Tumor Engagement & Checkpoint/Costimulation Targeting) platform, an innovative technology designed to deliver multifunctional tumor-specific activity while simultaneously enhancing immune modulation through the conditional activity of a natural immunomodulatory pair such as PD-1/PD-L1. The PROTECT mask employs a PD1/PD-L1 protein pair, which sterically obstructs CD3 binding in peripheral tissues, thereby minimizing off-target T cell activation and associated systemic toxicity. Specifically, the PD-L1 moiety is fused to an anti-CD3 antibody via a linker sequence containing a protease-cleavage site. Upon cleavage in TME, the mask is removed, resulting in the formation of a trispecific antibody. This activated antibody provides distinct functionalities: TCE activity, checkpoint inhibition, and additional differentiated immune-modulatory capabilities. The universal applicability of the PROTECT PD-1/PD-L1-based masking strategy has been demonstrated across various TAAs, including HER2. This versatility underscores the platform’s potential to support the development of a broad range of targeted cancer immunotherapies (23).

Although significant progress has been made with protease-activated bsAb prodrugs, their efficacy depends on the presence and specificity of tumor-associated proteases, as well as the expression of the corresponding tumor antigen by the target cell. A major challenge remains in designing protease-activating sequences uniquely specific to tumor-associated proteases, which must be localized exclusively to the tumor and show no activity in systemic circulation (24).

### 2.2 Proteolytic release of steric hindrance

The interaction between enzymes and their specific antibodies has been found to reduce enzyme activity (25). This inhibition mechanism seldom involves direct antibody binding to the catalytic site; instead, steric hindrance typically prevents substrate entry into the active site (25). In some systems, antibody interactions induce conformational changes in enzymes, which may either inhibit or enhance their catalytic activity. Common proteolytic enzymes in TME include MMPs, urokinase-type plasminogen activator (uPA), and ADAM proteins. It has been demonstrated that hemagglutinin stalk-reactive monoclonal antibodies can interfere with influenza virus neuraminidase activity via steric hindrance (26).

Silke Metz and colleagues reported a bsAb prodrug that relies on the proteolytic cleavage of linker peptides to remove steric hindrance within the antibody structure. This bsAb targets HER3 in a bivalent IgG-like configuration and incorporates an additional binding entity, anti-c-MET, consisting of a variable heavy (VH) and variable light (VL) domain linked by a disulfide bond. The trivalent, IgG-shaped molecules include a disulfide-stabilized Fv (dsFv) as a third binding entity, which lacks a linker between its VH and VL domains. Tethering these domains to the C-terminus of the CH3 domain reduces the dsFv’s on-rates for targeting antigens without affecting off-rates (restricted dsFvs moieties). Proteolytic cleavage of one of the two linked peptides alleviates this steric hindrance, effectively ending the ‘restricted binding mode’ (**Figure 1B**). This ‘release’ process significantly restores the affinity of dsFv, allowing it to exhibit full flexibility and rotational freedom around the remaining single flexible connector (27).

### 2.3 Soluble factor-dependent activation

The pathological proliferation of malignant cells induces significant alterations in the TME, distinguishing it from normal tissues in various aspects. Altered metabolic activity, uncontrolled proliferation, and elevated cell death lead to hypoxia, increased protease expression and activation, elevated lactate levels and low pH, high extracellular ATP, redox imbalances, increased cell-free DNA, and other altered soluble factors (28). These TME characteristics have been leveraged to achieve tumor-selective “conditional activation” of bsAbs, enhancing therapeutic efficacy and minimizing side effects (29).

The concept of soluble factor-dependent activation was initially demonstrated with monospecific antibodies. STA551, a novel anti-CD137 agonist switch antibody, binds to the extracellular domains of human and cynomolgus monkey CD137 in an ATP-, ADP-, and AMP-dependent manner. It activates CD137 selectively in the presence of ATP, inducing strong agonistic activity within the TME (30). Recently, this soluble factor-dependent mechanism has been extended to bsAbs. Bogen et al. developed a generic procedure to generate bsAbs with pH-sensitive binding modalities. This was achieved through modifications to the common light chain IGKV3-15*01 of a CEACAM5-targeting heavy chain-only antibody. One arm of the bsAb binds antigen in a pH-dependent manner, enhancing antigen (CEACAM5) clearance upon endosomal uptake. Meanwhile, the other arm retains pH-independent targeting of tumor cells. This class of pH-responsive bsAbs may be particularly effective for the efficient removal of soluble targets that promote tumor growth or suppress antitumor immunity (31). Thisted et al. further advanced this strategy by developing a novel CD28xVISTA co-stimulatory bsAb (BS2), engineered for tripartite ‘trans-activation’ of CD28 via pH-selective VISTA binding (**Figure 1C**). BS2 selectively acts in the acidic TME, inhibiting tumor growth *in vivo* and enhancing the activity of a CD3xPSMA bispecific T-cell engager (TCE) in human T-cell killing assays, with a reduced risk of cytokine release syndrome (CRS) (32). One persistent challenge lies in detecting the *in vivo* target binding of these switch antibodies. This challenge arises from the fact that switch antibodies may dissociate from the antigen during *in vivo* analysis, as ATP concentrations decrease due to enzymatic hydrolysis, spontaneous hydrolysis, or dilution. Therefore, observed *in vitro* binding may underestimate actual *in vivo* binding.

### 2.4 Conditional assembly on sites

Conditional assembly is another strategy for activating prodrugs in TME, where two independent molecules are assembled to exert their effects. This approach reduces systemic toxicity while enhancing the specificity of bsAbs. Baker and colleagues recently developed a system that addresses the challenge of optimizing bsAbs affinity, enabling the recognition of more complex antigen combinations. This system, termed LOCKR, comprises a ‘cage’ protein that encapsulates a functional peptide in its inactive state via the ‘Latch’ domain. Activation occurs only when the ‘Key’ protein binds, inducing a conformational shift that activates peptide binding to the “effector” protein. Studies demonstrate that the LOCKR system can precisely target CAR-T effector function without triggering off-target cell activation (33) (**Figure 1D**).

A T cell-engaging antibody derivative that comes in two complementary halves and addresses antigen combinations instead of single molecules. Each half, now called a hemibody, contains an antigen-specific scFv fused to either VL or VH domain of an anti-CD3 antibody. When two hemibodies simultaneously bind their respective antigens on a single cell, they align and reconstruct the original CD3 binding site to bind T cells (**Figure 1E**). It remains to be determined whether hemibodies will induce CRS (34). Revitope oncology is developing next-generation conditionally activated TCE, *Two*GATE, which consists of two bsAbs each targeting a distinct tumor antigen and forming an active anti-CD3 complex only when bound to the surface of a cancer cell (**Figure 1E**). Each of the CD3-targeting split paratopes is associated with a stabilizing domain and can only be activated by tumor-specific proteases. Leveraging *Two*GATE technology, Revitope is developing a pipeline of novel antibody prodrugs, such as REV-403, which targets EGFR and PD-L1.

A novel conditionally active TCE design, termed COBRA (Conditional Bispecific Redirected Activation) (35). COBRAs are designed to prevent CD3 binding and T cell activation until the scFv linker undergoes proteolytic cleavage. After cleavage, the inactive anti-CD3 scFv assembles to form active CD3 binding sites on the tumor cell surface (36). COBRA co-binds EGFR and CD3 only after dimerizing with a second fragment on the tumor cell surface (**Figure 1F**). When injected into mice with primary human T cells, COBRA with MMP2/9-cleavable linkers completely regresses established human tumors at modest doses. In contrast, COBRAs with non-cleavable linkers fail to induce tumor regression. This proteolysis-based strategy disrupts the binding conformation allosterically. Research demonstrated that COBRAs targeting HER2 exhibited antitumor efficacy, evidenced by tumor arrest or regression, and displayed the anticipated mechanism of action through T-cell extravasation into the TME (37). TAK-186 (also referred to as MVC-101) is a COBRA T-cell engager targeting EGFR and CD3, currently undergoing phase 1/2 clinical evaluation (NCT04844073) for patients with unresectable locally advanced or metastatic cancer (38). Like TAK-186, TAK-280 is a COBRA T-cell engager targeting CD3 and the B7-H3 protein, under phase 1/2 investigation (NCT05220098) in patients with solid tumors (**Table 1**) (39). A major challenge in conditional assembly lies in attaining balanced concentrations of COBRA components on tumor cell surfaces, which requires precise alignment of pharmacokinetic and pharmacodynamic properties for both molecules in individual patients. Additionally, achieving efficient large-scale production of monomeric COBRA components while mitigating premature aggregation in solution remains a significant obstacle in the absence of target-specific stabilization.

### 2.5 Chain exchange on target cells

Chain exchange represents a novel mechanism for activating bsAbs. Prodrug-Activating Chain Exchange (PACE) is based on the Format Chain Exchange (FORCE) technology, developed for robust, high-throughput generation of bsAb matrices *in vitro (* 40). PACE does not depend on proteolytic cleavage for activation; rather, it activates upon the simultaneous presence of two precursor molecules in close proximity. Chain exchange is driven by opposing charges introduced into the CH3 domains. This molecular architecture consists of a tumor-targeting Fab linked to a fully assembled, inactive Fab-like V-CH3 fusion, designed to prevent unpaired VH and VL domains from exposing hydrophobic interfaces, thereby improving the molecule’s biophysical properties. When the prodrugs encounter each other, such as within tumors, they exchange heavy chains, reconstituting active functional binders in TriFab formats. TriFabs are CH3-dimer-containing, IgG-derived bsAbs. They consist of two standard Fab arms for tumor targeting and a central third binding entity that replaces the regular CH2 domains (41). An intriguing feature of TriFabs is the vertical separation along the heavy chain interface, which splits the VH and VL domains of the Digoxigenin (Dig) binder into two inactive halves.

IL-4, a representative type I cytokine, was split and mutated to generate two inactive IL-4 prodrugs. IL-4 can be split into two topologies and fused to the C-terminus of antibody-like TriFab molecules. Pre-assembled split IL-4 retains activity but can be inactivated by introducing mutations into each half, generating inactive prodrugs (**Figure 1G**). Inactivation of split IL-4 prodrugs is maintained even after TAA-mediated accumulation on target cells, relying on chain exchange for reactivation. The expression yields of split IL-4 PACE prodrugs and pre-assembled split IL-4 products were significantly lower (4- to 40-fold) than PACE molecules without split IL-4. However, the expression yields of split IL-4 PACE molecules are consistent with previously reported levels for other antibody-cytokine fusions (42). A critical aspect of enhancing immunotherapy with cytokine prodrugs is ensuring a sufficient window between intended activation on target cells and unintended systemic activation.

## 3 Conclusions and future directions

Antibody prodrugs represent a novel class of antibody-based therapeutics with the potential to unlock effective biologic therapies previously hindered by target-associated toxicities. BsAbs enhance drug targeting by engaging multiple antigens simultaneously. By concentrating anti-tumor activity within the tumor TME, prodrugs may reduce severe toxicity and improve efficacy by enabling higher dosing frequencies. Although extensive preclinical and early clinical trial data are encouraging, the development of antibody prodrugs remains in its infancy. The ability to expand the therapeutic index of antibody prodrugs depends heavily on their design, though optimal design strategies are not yet fully elucidated. An additional challenge involves navigating the regulatory approval process. While regulators have expressed interest in advancing better-tolerated treatments, most antibody prodrugs remain under development or in early clinical evaluation.

A novel approach in cancer therapy involves the development and production of highly tumor-specific bsAbs termed prodrug-based bsAbs. These antibodies are activated by specific substances present within TME, offering a promising strategy to enhance therapeutic precision. This emerging technology demonstrates significant potential for future clinical applications. However, several critical challenges remain to be addressed. Firstly, compared to traditional therapeutic strategies, there is a notable lack of comprehensive clinical data and experimental validation for prodrug-based bsAbs (**Table 1**). This limitation highlights the need for rigorous preclinical and clinical studies to establish their safety and efficacy profiles systematically. Secondly, activation factors for prodrug-based bsAbs are not always exclusive to the TME, which increases the risk of premature activation in non-tumor tissues. Such off-target activation could lead to systemic toxicity, undermining the intended benefits. Therefore, improving the specificity of activation mechanisms is imperative to ensure selective tumor targeting while minimizing collateral effects. Lastly, the pharmacokinetics (PK) and pharmacodynamics (PD) of these prodrugs must be meticulously regulated. Optimizing these parameters is essential to achieve a balance between maximizing therapeutic efficacy and minimizing toxicity. Addressing these challenges will be critical for the successful translation of prodrug-based bsAbs into clinical settings.
